# Are heart toxicities in breast cancer patients important for radiation oncologists? A practice pattern survey in German speaking countries

**DOI:** 10.1186/s12885-017-3548-2

**Published:** 2017-08-23

**Authors:** Marciana Nona Duma, Stefan Münch, Markus Oechsner, Stephanie Elisabeth Combs

**Affiliations:** 1 Department of Radiation Oncology, Technical University of Munich (TUM), Munich, Germany; 2 Zentrum für Stereotaxie und personalisierte Hochpräzisionsstrahlentherapie (StereotakTUM), Technische Universität München (TUM), Munich, Germany; 3 Institute of Innovative Radiotherapy (iRT), Department of Radiation Sciences (DRS), Helmholtz Zentrum München, Munich, Germany; 4 Deutsches Konsortium für Translationale Krebsforschung (DKTK), Partnerstandort München, Munich, Germany

**Keywords:** Breast cancer, Pattern of care, Heart, Cardiac toxicities

## Abstract

**Background:**

To assess the personal beliefs of radiation oncologists regarding heart sparing techniques in breast cancer patients.

**Methods:**

Between August 2015 and September 2015, a survey was sent to radiation oncology departments in Germany, Austria and Switzerland. 82 radiation oncology departments answered the questionnaire: 16 university clinics and 66 other departments. Most (87.2%) of the participants had >10 years of radiation oncology experience.

**Results:**

89.2% of the participants felt that there is enough evidence to support heart sparing for breast cancer patients. The most important dose parameter was considered the mean heart dose (69.1%). The personal “safe” dose to the heart was considered to be 5 Gy (range: 0–40 Gy). The main impediment in offering all breast cancer patients heart-sparing techniques seems to be the fact that these techniques are time/ resource consuming (46.5% of the participants).

**Conclusions:**

Most radiation oncologists believe that there is enough evidence to support heart sparing for breast cancer patients. But translating this belief into a wide practice will need better dosimetric and clinical data on what patients are expected to profit most, specific guidelines for which patients’ heart sparing techniques should be performed, as well as recognition of the time/resource consumption of these techniques.

## Background

Large retrospective data have demonstrated a relationship between the delivered heart dose and major coronary events in breast cancer radiotherapy [1–5].

Thus, dose constraints to the heart and coronary arteries have become important in the treatment planning process.

Today, different heart sparing techniques are used in the clinical routine. As highlighted by as Shah et al. [6] these techniques can be broadly divided into three categories:

1. maneuvers that displace the heart from the irradiation field such as coordinating the breathing cycle or through pronepositioning,
2. technological advances such as intensity modulated radiation therapy (IMRT) or volumetric modulated radiation therapy and
3. techniques that treat a smaller volume around the lumpectomy cavity such as accelerated partial breast irradiation (APBI), or intraoperative radiotherapy (IORT).

However in which extent these techniques are used for breast cancer patients in the clinical routine is still unknown. Many radiation oncologist claim they use all abovementioned techniques, and scientific discussions are ongoing. However, no data is available on what the real clinical reality is, and which approaches are used in daily routine. Thus, the aim of our survey was to perform a practice pattern survey in the German speaking countries mainly focused on the practical application of heart sparing techniques. This paper focusses on the personal beliefs of radiation oncologists, which dosimetric data to the heart are clinically meaningful and, based on all data available, which dose-reduction strategies are ready for clinical routine application. The available literature is discussed, especially with regard to the heart structures that should be contoured during treatment planning and the dose that should be accepted during treatment planning.

## Methods

Between August 2015 and September 2015, an email/fax based survey was sent to radiation oncology departments in German speaking countries. Ethics approval by our committee was not applicable for a pattern of care study involving online questionnaires sent to radiation oncologists. To generate the questionnaire we collected all items relevant to the topic. We then formulated the questions and performed a test-phase within radiation oncologist in our department. With this we checked whether questions were understandable, the answers were easy to choose, and whether any important information was missing. After validation within this cohort the questionnaire was adapted and then sent out to the whole test population. Internal consistency was tested through the extensive validation within a group of experienced radiation oncologist reviewing the questionnaire and collecting any missing items. 82 radiation oncology departments answered the questionnaire: 16 university clinics and 66 other departments. The questionnaire was divided into 3 chapters: a general chapter on the department, a chapter specific for heart sparing techniques in breast cancer patients [7] and a third chapter on personal beliefs on the topic of heart sparing. In this paper we will focus on the personal beliefs of the radiation oncologists, correlated to the actual situation in the departments.

The third part consisted of 12 questions (Table 1). Questions 6, 7, 8 and 9 were multiple choice questions. The questionnaires returned were evaluated anonymously using the SPSS statistical program (version 23, IBM SPSS Statistics).

**Table 1:** Questionnaire

Question number	Question	Possible answers:
1.	Age	____ years
2.	Sex	• Male • Female
3.	Radiotherapy experience	____ years
4.	Do you feel that there is enough evidence to support heart sparing for breast cancer patients?	• Yes • No • I don’t know
5.	Which dose do you consider a “safe” heart dose?	________ Gy
6.	Which patients do you think will profit from heart sparing techniques?	• all patients • patients who underwent cardiotoxic systemic therapy • patients with known arterial hypertension • patients with known coronary heart disease • patients < 50 y.o • patients <60 y.o. • patients <70 y.o. • patients <80 y.o. • patients <90 y.o. • other (please specify):_______
7.	Which dosimetric parameter do you consider most important?	• V10 (the volume that receives 10 Gy or more) • V20 • V30 • V40 • V50 • Dmean • Dmax • other (please specify):_______
8.	For which structures are the previously chosen parameters important for you?	• whole heart • left anterior descending artery • right coronary artery • ramus circumflexus • left ventricle • right ventricle • left atrium • right atrium • other (please specify):_______
9.	You would offer heart sparing techniques to all breast cancer patients if:	• It would be less time/ resource consuming. • There would be better evidence in literature. • I could decide by myself. • The reimbursing would be better. • I would not offer heart-sparing techniques to all patients. • other (please specify):_______
10.	Do you feel that there is enough evidence to support heart sparing for other cancer patients (e.g. Hodgkin lymphoma)?	• Yes • No • I don’t know
11.	Do you perform heart sparing for other cancer patients (e.g. Hodgkin lymphoma)?	• Yes • No
12.	If yes, for which entities?	_________________

## Results

The overall return rate was 40%, with 55% return rate of the university hospitals. The median age of the radiation oncologists that answered the questionnaire was 48.5 years (range 29 years to 65 years). 51 participants (62%) were male. The median radiotherapy experience of the participants was 20 years (minimum 4 years and maximum 35 years). 89.2% participants considered the evidence for heart sparing in breast cancer patients as sufficient. The majority of participants (57.9%) deemed age an important selection criterion for heart sparing. Of these 20.3%, 15.9% and 21.7% felt that in order to benefit from heart sparing radiotherapy the patients should be younger than 50 years, 60 years and 70 years, respectively. The remaining 40.6% didn’t regard age as criterion for heart avoidance. 84.5% think that the patients with known cardiovascular disease would profit from heart sparing.

**Fig. 1:**
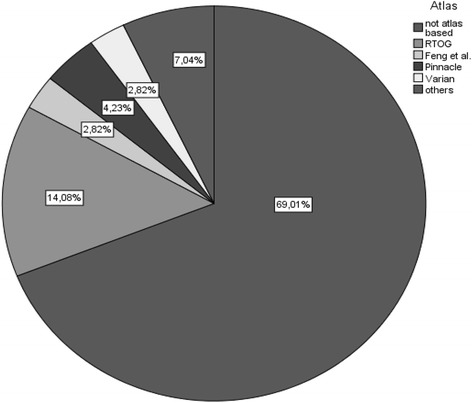
Atlas based contouring of the heart during routine treatment planning. “others” included several different diagnostic CT atlases available in the departments

The most frequent answer to the question “How many of your breast cancer patients undergo heart sparing radiotherapy?” was “25%–50% of the patients” (41.5% of the departments), followed by “<25% of the patients” (28.0% of the departments). But, on the other hand, 53.7% of the departments did not have written institutional guidelines when heart sparing techniques should be performed. 69.0% of the departments did not perform an atlas based contouring of the heart. If atlas based contouring is performed, the most often used atlas was the Radiation Therapy Oncology Group (RTOG) thorax atlas (with 14.1%) (Fig. 1).

**Fig. 2:**
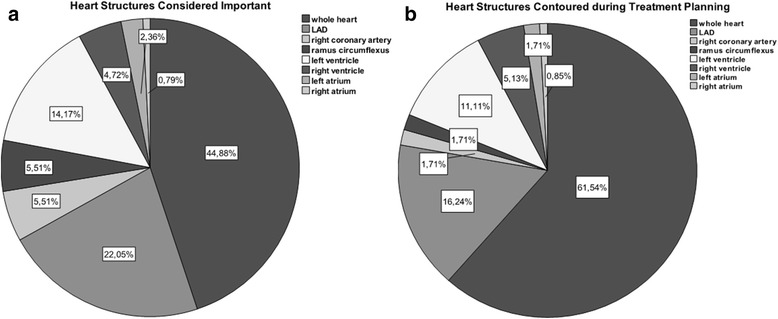
Heart structures that are routinely contoured during treatment planning (**a**) as opposed to structures that are considered important for heart toxicities (**b**)

Figure 2 depicts the structures of the heart that are contoured during treatment planning and the structures personally considered important.

The most important dosimetric parameter was considered the Dmean (by 69.1% of the participants). The other dosimetric parameters considered important were (in descending order): the Dmax (29.6% of the participants), the V10 (19.8%), V30 (16.0%), V20 (12.3%), V40 (9.9%) and V50 (8.6%). For the departments, that had written institutional guidelines, the median Dmean dose threshold was 3 Gy (range 2–25 Gy). The median “safe” dose to the heart was considered to be a Dmean of 5 Gy, with a range 0–40 Gy.

The main impediment in offering all breast cancer patients heart sparing techniques seems to be for almost half (46.5%) of the participants the fact that these techniques are time/resource consuming. The other main reasons were in descending order: “There would be better evidence in literature” (25.7%), “The reimbursing would be better” (15.7%) and “I could decide by myself.” (8.6%).

**Fig. 3:**
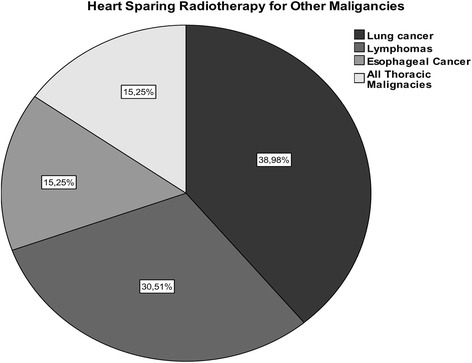
Heart sparing radiotherapy offered for other malignancies than breast cancer

94.2% of the participants feel that there is enough evidence to support heart sparing for other cancer patients too, and 61.0% perform heart sparing for other cancer patients. Figure 3 depicts the entities.

## Discussion

Heart sparing in breast cancer patients seems to be an important issue for most radiation oncologists according to our survey, however they do not use it for all patients. Why is that? There seem to be two main problems that should be solved and must be addressed in further scientific work:

1. which are the relevant heart structures and how should the contouring of the heart and heart subvolumes be performed;
2. what is a “safe” dose and is the Dmean to the whole heart the best dosimetric parameter?

Three late toxicities are described after breast cancer radiotherapy: myocardial infarction/ischemic heart disease; congestive heart failure and valvular diseases. Retrospective studies have demonstrated that after left sided breast cancer radiotherapy, in patients with ischemic heart disease most abnormalities at stress tests and catheterizations were found in the left anterior descending artery (LAD) [8, 9]. Congestive heart failure is mainly related to microvascular damage (decrease in capillary density) that lead to interstitial myocardial fibrosis. Several studies demonstrated that perfusion defects after breast cancer radiotherapy appear to be related to the irradiated volume of the left ventricle and largely persists for many years after radiotherapy [10–12]. The pathogenesis of valvular damage by radiotherapy in breast cancer is still not well understood, but it was mostly correlated with the irradiation of the internal mammary chain [13, 14].

Thus, three heart structures seem to be important and should be contoured during treatment planning: the coronary arteries (especially the LAD), the myocardium and the valvular system. However, large interobserver contouring variability of these structures is documented. In an older study by the RTOG, contouring uncertainties and the inherent dosimetric uncertainties have been found to be clinically significant and the need for a standardized approach was postulated [15]. Nowadays, several contouring atlases are available [16, 17]. But, even atlases cannot overcome certain contouring uncertainties. Lorenzen et al. found substantial inter-observer variation for the delineation and the estimated dose of the LAD, which even guidelines could not reduce [18]. The coefficients of variation in the estimated doses to the LAD were for the mean dose 27% without and 29% with guidelines. For the heart, variations were little, especially when guidelines were used [18]. Contouring of the myocardium or the valvular system is hindered mostly by planning CTs that are routinely performed without contrast medium.

The answer to the question what the “safe” heart dose in breast cancer is still not known [2] as there are few available studies on CT derived doses and correlations to late toxicities. A recent study provides some help how 2 D simulator films might be used for estimating mean doses to the whole heart in left-tangential radiotherapy for breast cancer and might enhance our knowledge on this issue [19]. No large studies are available in breast cancer that correlated clinical outcomes to CT derived individual doses to substructures of the heart – i.e. the coronary arteries, the myocardium or the valvular system. Despite correlating solely the dose recalculated on a “typical” patient to the incidence of major coronary events, the paper by Darby et al. is a landmark in this field of clinical research [1]. It provides an estimation of risks taking the Dmean to the heart into account. The study states that the mean dose of radiation to the heart was a better predictor of the rate of major coronary events than the mean dose to the LAD. This is not surprising, as the LAD contouring uncertainties are high, and the contouring for this study was not done individual for every patient. Nonetheless, if we suppose we treat a cardiac healthy 40-year-old woman with a Dmean to the heart of 5 Gy (the median “safe” dose in our practice pattern survey) her risk of having at least one acute coronary event by the age of 80 increases from 4.7% to 6.4%^1^. If the same 40-year-old woman would have at least one cardiac risk factor, the increase would be from 7.9% to 10.7%. Further, the study postulated an increase of major coronary events by 7.4% per gray (mean dose to the heart). Sardaro et al. postulated an increase of 4% per gray [20].

There are no clinical studies that performed correlations of the Dmean to the heart to the Dmean to the left ventricle or the valvular system in breast cancer and correlated them to long-term toxicities [21–23].

The delineation/dose/toxicity issue is further complicated by different fractionation schedules (normo- vs. hypofractionation) as well as combination of systemic therapy [24, 25].

Major arguments against the use of heart sparing techniques were time-consuming setup and treatment times, which are not reflected in reimbursement codes. Knowing that these arguments should not impair high-end patient treatment, however, it should be kept in mind that especially for smaller institutions with limited time and money resources these arguments could be of high importance. Therefore, advanced techniques must be reflected in modern reimbursement codes.

To sum up, three structures - the coronary arteries, the myocardium and the valvular system- are pathophysiologically important. These structures should be contoured during the treatment process. However, definite dose constraints cannot be defined with the available data. The Dmean to the whole heart seems to be a good surrogate for the toxicities related to the coronary arteries (i.e. major coronary events). We have no evidence that there is a “safe” heart dose. Thus, the decision what “risk” is acceptable is left to the clinical judgment of the treating radiation oncologist.

## Conclusions

Our pattern of practice survey demonstrated that most radiation oncologists believe that there is enough evidence to support heart sparing for breast cancer patients and some departments have implemented this into the clinical routine for almost half of their patients. Translating this belief into a standardized clinical practice will need better dosimetric and clinical data on what patients are expected to profit most, specific guidelines for which patients’ heart sparing techniques should be performed and how the contouring should be done, as well as a recognition of the time/resource consumption of these techniques.

## Abbreviations

APBI: Accelerated partial breast irradiation
IMRT: Intensity modulated radiation therapy
IORT: Intraoperative radiotherapy
LAD: Left anterior descending artery
RTOG: Radiation Therapy Oncology Group
